# Vector nematodynamics with symmetry-driven energy exchange

**DOI:** 10.1140/epje/s10189-026-00600-z

**Published:** 2026-06-17

**Authors:** L. M. Pismen

**Affiliations:** Department of Chemical Engineering, Technion–Israel Institute of Technology, Haifa, 32000 Israel

## Abstract

**Abstract:**

We review inadequacy of existing nematodynamic theories based on Onsager’s near-equilibrium relations and suggest a novel way of establishing relations between nematic orientation and flow, based on the *local* symmetry between simultaneous rotation of nematic alignment and flow, which establishes energy and momentum exchange between the two without reducing the problem to near-equilibrium conditions. This approach, applied in the framework of the vector-based theory with a variable modulus, involves antisymmetric interactions between nematic alignment and flow. It avoids spurious instabilities in the absence of active inputs and elucidates their cause.

**Graphical abstract:**

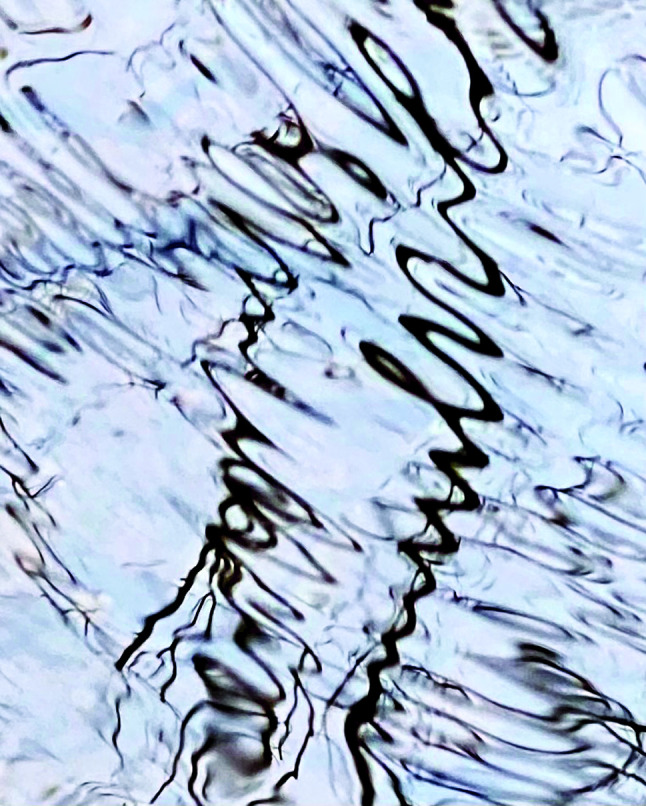

## Why nematodynamic theory needs renewal

The renewed interest in dynamics of nematic fluids has arisen owing to its wide applications in studies of active matter. Nematic order is commonly encountered in biological tissues [1–3], cells [4, 5], and bacterial swarms [6, 7] colonies [8], and biofilms [9]. In all these systems, the constitutive elementary units are macroscopic, in contrast to molecular units of common nematic liquids. Nevertheless, theories of active nematic are commonly based on the established nematodynamic theory supplemented by a phenomenological active input. While the analysis of specific problems of active matter that would require a revaluation in the light of the suggested approach is beyond the scope of this communication, it is expected that it will induce interested researchers to reexamine relevant topics.

The established theories aspire to be universal: they do not assume any specific mechanism of interactions between rearrangements of nematic order and flow, but derive dynamics close to equilibrium by assigning general linear relationships between thermodynamic forces and fluxes and establishing relations between their coefficients via Onsager’s reciprocal relations. The original Ericksen–Leslie (EL) approach [10–12] employing the nematic director as the order parameter, has been later extended to allow for a variable modulus through the use of the tensor order parameter [13, 14], but all these theories, briefly reviewed below, rely on near-equilibrium relations between thermodynamic forces and fluxes. There is a general objection to this approach [15] stating that a combination of variables even and odd under time reversal is *physically unsound* and cannot ensure evolution to thermodynamic equilibrium, but it has been generally ignored, even after it has been shown by straightforward computation [16, 17] that instability may arise under certain conditions in nematodynamic equations derived in this way even in the absence of an active input, which is ostensibly forbidden by Onsager’s relations.

The paper is organized as follows. First, the theory of “dry” nematics is reiterated in Sect. 2, following its development from the director- to tensor-based description and paying a particular attention to the less common vector-based description which, though being equivalent to the tensor-based one, is more convenient for the description of the novel approach based on the local symmetry between rotation of the nematic alignment and flow in Sect. 3.1, which is the central point of this communication. The following Sect. 3.2 is dedicated to a more detailed analysis of the energy and momentum exchange between nematic alignment and flow, including the antisymmetric stress and viscous anisotropy. Finally, Sect. 4 presents the stability analysis of the ordered state and explains the source of spurious instabilities in the absence of active inputs detected earlier.

## “Dry” nematodynamics

### From director to tensor order parameter

The classical director-based description is deficient even in “dry” problems lacking hydrodynamic or mechanical interactions. The director $\textbf{n}$ is formally a unit vector, but, unlike proper vectors, it is supposed to be symmetric to rotation by $\pi$, and therefore any energy expression should include an even power of $\textbf{n}$. Deviations of a static nematic medium from equilibrium may be caused in this framework only by changes in orientation expressed by differential terms dependent on distortions of perfect alignment. The classical lowest-order expression for the energy density of a uniaxial nematic in 3D, the simplest form of the Landau–de Gennes Lagrangian going back to Frank [18], is

1 $$\begin{aligned} \mathcal {L}&=\textstyle \frac{1}{2}\bigg [ K_1( \textrm{div}\, \textbf{n})^2 + K_2 ( \textbf{n} \cdot \textrm{curl}\, \textbf{n})^2 \nonumber \\&\quad + K_3 ( \textbf{n} \times \textrm{curl}\, \textbf{n})^2 \bigg ]. \end{aligned}$$

The three terms in this expression correspond to the energies of splay, bend, and twist distortion of nematic alignment. They include all possible quadratic combinations of the director with its divergence and curl invariant to rotations by $\pi$. The coefficients $K_i$, called Frank constants, are the respective elasticities.

The dynamic equation that defines evolution of the nematic director in a quiescent medium, obtained by varying the Lagrangian (1), is $\mathcal {D}_t \textbf{n}_j=\Gamma \textbf{h}_j$, where $\mathcal {D}_t=\partial _t + \textbf{v}_0\cdot \nabla$ denotes the substantial derivative accounting for advection with a *constant* flow velocity $\textbf{v}_0$, according to Galilean invariance. The mobility coefficient $\Gamma$ is commonly assumed to be a scalar. The evolution is driven by the *molecular field* $\textbf{h}$ characterizing orientational distortions:

2 $$\begin{aligned} h_i =\partial \mathcal {L}/\partial n_i + \partial _j \pi _{ji}, \quad \pi _{ji}= \partial \mathcal {L} /( \partial _jn_i). \end{aligned}$$

Summation over repeated indices is implied throughout.

Generally, $\mathbf {n\cdot h}\ne 0$, so that the normalization of $\textbf{n}$ is not preserved, but it is sometimes sustained, without any physical justifications, by adding to (1) a Lagrange multiplier. Of course, the normalization $|\textbf{n}|=1$ cannot be preserved in real nematic textures; for example, $\textbf{n}$ vanishes or becomes indefinite in the core of topological defects. Nevertheless, this approach has not been so far abandoned, and, unfortunately, the most commonly read and cited textbook on nematodynamics [12] does not go beyond the director-based description.

Realistic theories with a variable modulus are commonly based on the tensor representation [13, 14, 19]. The tensor order parameter, commonly expressed for uniaxial nematics in *d* dimensions by the symmetric traceless tensor $Q_{ij}= (n_in_j-\delta _{ij}/d)$ supplemented by the modulus $\varrho$. It involves coupled inclination angles, and therefore is invariant with respect to rotation by $\pi$. The tensor description works perfectly in “dry” nematodynamics, although there are some problems with retaining all Frank constants while keeping the lowest-order Lagrangian [17].

The “wet” problem is not better settled in the realistic tensor-based than in the obsolete director-based theory. There are two distinct versions [19], and, while the Harvard Theory is more extensive, being applicable beyond uniaxial nematics, the authors warn that they “consider only small deviations from a state in equilibrium and at rest” and admit that “the equations proposed by Leslie and Ericksen are also equivalent to the present result.” [20]. Therefore we concentrate here on the criticism of the more influential EL approach, which has been already questioned [15] on the basis of an improper use of Onsager’s reciprocity relations due to combining fields with opposite time-reversal symmetry.

### Vector-based description

The progress from director-based to tensor-based theory has skipped an intermediate possibility—vector-based description, but it has been introduced not long ago as a 2D “nemator” [21] and independently explored by this author [16, 17]. Two-dimensional patterns are commonly observed in thin layers with tangential alignment on containing walls, as well as in thin elastic sheets and cellular layers, and computational models are commonly restricted to 2D, as 3D simulations are too cumbersome and hard to present on a 2D screen or paper sheet.

In 2D, it is sufficient to characterize the nematic alignment by the vector order parameter $\textbf{q}$ with the components

3 $$\begin{aligned} q_1= (n_1^2-n_2^2)= \cos 2\theta , \;\; q_2=2 n_1n_2 =\sin 2\theta . \end{aligned}$$

By construction, $|\textbf{q}|=1$, and, as in the tensor-based theory, it should be complemented by the modulus $\varrho$. Similar to the 2D tensor $\textbf{Q}$, $q_i$ are invariant to rotation by $\pi$, and $\textbf{Q}$ can be presented as a merger of $\textbf{q}$ and its rotation by $\pi /2$, $\widehat{\textbf{q}}$ with the components $\widehat{q}_i=\varepsilon _{ij}q_j$, where $\varepsilon _{ij}$ is the 2D Levi-Civita antisymmetric symbol. This leads to

4 $$\begin{aligned} \textbf{Q}=\textbf{q}\widehat{\textbf{q}} =\begin{pmatrix} q_1 &  \widehat{q}_1 \\ q_2 &  \widehat{q}_2 \end{pmatrix} =\begin{pmatrix} \cos 2\theta &  \sin 2\theta \\ \sin 2\theta &  - \cos 2\theta \end{pmatrix}. \end{aligned}$$

The vector-based analog of the Lagrangian (1) can be constructed by imitating the latter’s general form with the twist term omitted in 2D, while replacing $\textbf{n}_i$ by $\textbf{q}_i$ and adding the algebraic term defining the modulus:

5 $$\begin{aligned} \mathcal {L}=\alpha -\varrho ^2+\mathcal {L}_q, \end{aligned}$$

where $\alpha$ is the bifurcation parameter defining the transition to an anisotropic state at $\alpha >0$. The dynamic equation of the order parameter is constructed in the same way as in Eq. (2) with $\textbf{n}$ replaced by $\textbf{q}$:

6 $$\begin{aligned} {h}_i =\partial \mathcal {L}/\partial {q}_i + \partial _j \pi _{ji}, \quad \pi _{ji}= \partial \mathcal {L}_q /( \partial _j{q}_i). \end{aligned}$$

The explicit 2D expression is

7 $$\begin{aligned} \boldsymbol{\pi } = \begin{pmatrix} P_1 &  P_2 \\ -P_2 &  P_1\end{pmatrix}\; \textrm{with}\; \begin{pmatrix}P_1=K_1(\partial _1 {q}_1+ \partial _2 {q}_2) \\ P_2 =K_2(\partial _2 {q}_1- \partial _1 {q}_2)\end{pmatrix}. \end{aligned}$$

This representation can be extended (for a uniaxial nematic) to 3D ensuring, as in 2D, the invariance to rotation by $\pi$ by coupling director components. The expression constructed in this way, $\textbf{q}^{(i)}=\varepsilon _{ijk}n_j n_k$ (where $\varepsilon _{ijk}$ is the 3D Levi-Civita antisymmetric symbol), can be specialized in different ways invariant to rotation by $\pi$. The form most suited to the above 2D expression is constructed by defining the components $q_j, \,q_k$ as a 2D vector with the components $\cos 2\theta _{jk},\, \sin 2\theta _{jk}$, where $\theta _{jk}$ is an angle in the plane normal to $n_i$ (which is invariant to the latter’s reversal).

The lowest-order distortion-dependent part of the Lagrangian $\mathcal {L}_q$ representing the elastic energy per unit volume can be constructed in alternative ways, out of which we choose the one most similar by its structure to its director-based analog (1):

8 $$\begin{aligned} \mathcal {L}_q&= \textstyle \frac{1}{2} \left\{ K_1[\textrm{div}\, \textbf{q}]^2 + K_2 [q_i \cdot q_j \times q_k)]^2 \right. \\&\quad +\left. K_3 [q_i \times (q_j \times q_k)]^2\right\} . \end{aligned}$$

This construction can be extended to biaxial nematics by supplementing the three parameters defining the inclination of the main axis by a 2D vector characterizing the projection of a secondary director on the plane normal to the primary one.

Although the vector-based description is physically

equivalent to the tensor-based one, it allows an easier construction of the Lagrangian and more freedom in its adjustment to specific problems by choosing a particular direction $q_i$ in the general expression (8). Moreover, if there exists a prevailing “reference” orientation $\textbf{q}^0$, the 3D Lagrangian can be simplified by redefining $q_i$ as *deviations* from this orientations:

9 $$\begin{aligned} \mathcal {L}_q&= \textstyle \frac{1}{2} \left[ K_1( \textrm{div}\, \textbf{q})^2 + K_2 (\textbf{q}^0 \cdot \textrm{curl}\, \textbf{q})^2 \right. \\&\quad \left. + K_3 ( \textbf{q}^0 \times \textrm{curl}\, \textbf{q})^2 \right] . \end{aligned}$$

While in the framework of “dry” nematodynamics, the usage of the vector order parameter enables applying the classical formalism to systems with a variable modulus with minimal adjustments, it does not help a harsher problem: the inadequacy of applying Onsager’s relations for derivation of a complete set of nematodynamic equations coupling nematic reorientation and flow. This procedure involves special “alignment”, or “tumbling” parameters introduced to accommodate required symmetries. Instabilities, forbidden in passive systems, have been observed within some range of the latter’s values [16, 17], suggesting that substantial changes of the approach are necessary.

## Vector nematodynamics

### Interactions induced by rotational symmetry

The primary cause of the change of nematic orientation is the tendency to ordering, characterized by the molecular field defined in the vector-based theory by Eq. (6), which can be extended in 3D to a triple expression dependent on the choice of a base direction $q_i$. A complementary course of reorientation, overlooked by extant theories, is rotation of nematic alignment by local flow. It follows from the *local* symmetry between simultaneous rotation of nematic alignment and flow:

10 $$\begin{aligned} \partial _t n_i= A^-_{ij}n_j, \quad \partial _t x_i=A^-_{ij}x_j, \end{aligned}$$

where $A^-_{ij}$ is the antisymmetric part of the rate of strain tensor $A^\pm _{ij}=\textstyle \frac{1}{2} (\partial _iv_j \pm \partial _jv_i)$. Since $\textbf{q}$ rotates with twice director’s speed, the vector-based energy-conserving relation is written as

11 $$\begin{aligned} \partial _t q_i= 2A^-_{ij}q_j, \quad \partial _t x_i=A^-_{ij}x_j. \end{aligned}$$

Although this symmetry affects the tensor $\textbf{Q}$ as well, it is less conveniently expressed in its common representation as a traceless matrix, which formally (but not in substance) differs from Eq. (4).

In standard theories, this symmetry is viewed as *global*, which conceals its role in the exchange between elastic and hydrodynamic energy. The vector

12 $$\begin{aligned} \boldsymbol{\varpi }=2\textbf{A}^- \cdot \textbf{q} \end{aligned}$$

represents the rate of rotation of the order parameter by fluid flow, lowering the latter’s energy, as distinguished from rotation driven by lowering the elastic energy. Thus, the total rotation is computed as

13 $$\begin{aligned} \partial _t q_i= \Gamma \textbf{h}_i+\boldsymbol{\varpi }_i, \end{aligned}$$

while the vector $\boldsymbol{\varpi }$ enters the hydrodynamic energy balance, as discussed below.

A so far overlooked aspect of the rotational symmetry is its role in nematodynamic energy and momentum balance. While rotation driven by reducing nematic energy generates the distortion stress $\sigma ^\textrm{d}_{ij}=-\pi _{kj}\partial _i q_k$, the symmetry-driven rotation is purely hydrodynamic and induces the antisymmetric viscous stress, overlooked both by director- and tensor-based theories and taken in account so far only in this author’s study of a specific model [22].

### Energy and momentum exchange

Elasto-hydrodynamic interactions involve exchange between the elastic energy $\mathcal {E}= \int \mathcal {L}\,\textrm{d}\textbf{x}$ and flow energy

$\mathcal {F}=\textstyle \frac{1}{2}\int \rho |\textbf{v}|^2\textrm{d}\textbf{x}$. The latter’s change with time depends on acceleration of the fluid determined by the generalized Navier–Stokes (NS) equation $\rho \mathcal {D}_t v_i = f_i$, where $\rho$ is the fluid’s density and $\textbf{f}({\textbf {x}})$ is the total force acting upon the fluid. In the standard hydrodynamics of isotropic fluids, this force includes the pressure gradient $\nabla p$ and the symmetric viscous stress $\boldsymbol{\sigma }^+$, dependent linearly on the symmetric rate of strain $\textbf{A}^+$. In an oriented fluid, this relation is generally anisotropic, $\sigma ^+_{ij} = \eta _{ijkl}^+A^+_{kl}$, with viscosities $\eta ^+_{ijkl}$ expressed as a fourth-order tensor built up of combinations of $q_i$ (or $n_i$ in the EL theory) with empirical coefficients.

The anisotropy of the symmetric stress–strain relation can be included by incorporating the order parameter $\textbf{q}$ in the same way as the director is incorporated in the EL theory [12]. In this way, five symmetric viscosities are defined:

14 $$\begin{aligned} \sigma ^s_{ij}&= \eta _1^sA^+_{ij} +\textstyle \frac{1}{2}\eta _2^sq_k( q_iA^+_{kj}+q_jA^+_{ki}) \\&\quad {+}\eta _3^s q_iq_jq_kq_lA^+_{kl}{+}\eta _4^s \delta _{ij}A^+_{kk} {+}\eta _5^s q_iq_jA^+_{kk}. \end{aligned}$$

The antisymmetric stress can expressed in a similar way but with $\textbf{A}^+$ replaced by $\textbf{A}^-$, $\eta _i^s$ by $\eta _i^a$ and the last two terms excluded.

The EL theory includes a larger number of independent anisotropic viscosities constructed by including “tumbling”, or ”alignment”, parameters. These parameters are irrelevant in the framework of the theory presented here, as they have nothing to do with actual flow-induced alignment (Sect. 3.1) but are just artifacts serving to ensure symmetry between particular terms in the relations between generalized Onsager’s “forces” and “fluxes” in the EL approach.

In the framework of the present theory, the hydrodynamic force related to the change of nematic orientation originates in the flow energy driving the rotation of the order parameter, as given by the first relation (11). Rotation costs energy, and is counteracted by viscosity, generally anisotropic, generating the antisymmetric stress $\sigma ^-_{ij} =-2\eta _{ijk}^-A^-_{kl}q_l$ with an antisymmetric (odd) viscosity $\eta _{ijk}$ dependent on local orientation. This relation, alongside the velocity-dependent Eq. (13), carries the connection between flow and elastic dynamics. The simplified isotropic version of the odd viscosity is $\eta ^-_{ijk}=\delta _{ij}\eta ^-_k$. The simplified version of the antisymmetric odd viscosity is the vector $\eta ^-_{k}$.

Neglecting viscous anisotropy, the applicable *linear* hydrodynamic balance (with the convective time derivative replaced by the simple one) is expressed as

15 $$\begin{aligned} \rho \partial _t v_i=\partial _k(\eta ^+ A^+_{ik} -2 \eta ^-_a q_aA^-_{ik}) -\partial _i p. \end{aligned}$$

The NS equation is commonly complemented by the gradient of the distortion stress $\boldsymbol{\sigma }^\textrm{d}$ stemming from the reduction of elastic energy. It plays a secondary role in the analysis, being quadratic in small perturbations, e.g., the 2D expression following from Eq. (7) is

16 $$\begin{aligned} \boldsymbol{\sigma }^\textrm{d} = - \begin{pmatrix} P_1 \partial _1 P_1- P_2 \partial _2 P_2&  P_2 \partial _1 P_1+ P_1 \partial _1 P_2\\ P_1 \partial _2 P_1- P_2 \partial _2 P_1 &  P_2 \partial _2 P_1+ P_1 \partial _2 P_2\end{pmatrix}. \end{aligned}$$

Inclusion of this stress is questionable in the framework of the present approach, since the energy released due to nematic ordering may be dissipated as heat rather than affecting directed fluid motion. The clear-cut case of the momentum transfer is that due to the local symmetry, the principal topic of this communication, but it does not provide a way to estimate the balance between the distortion stress and heat dissipation, and this problem requires experimental verification.

The total energy or enthalpy must decrease in a system evolving to an equilibrium or a stable stationary state. Unlike all extant theories, this criterion does not rely on Onsager’s relations and can be applied as well to active systems driven from equilibrium by a steady active input and evolving to a stable non-equilibrium state. Of course, implementing this criterion requires finding both the order parameter and flow velocities by solving the dynamic equations for the order parameter and velocity, and only stability of an ordered state to *weak* perturbations can be established analytically (see the next Section). Unfortunately, the role of nonlinear anisotropic viscous terms, as well as of the distortion stress, cannot be estimated in this way.

## Stability of a quiescent state

As a basic example of application of the linear stability analysis, we consider perturbations of an ordered state in an unbounded domain. The simplest case is a 2D problem with the original orientation along the $x_1=x_\Vert$ axis, realizable in an adsorbed layer or a thin sheet with planar orientation imposed by boundary conditions on confining walls. This approach can be extended to 3D in the cylindrical geometry where rotational velocity is irrelevant for deviations of the alignment from the symmetry axis.

The standard procedure of the linear stability analysis is based on expanding virtual deviations from a stationary ordered state in small parameter $\epsilon \ll 1$. An $\mathcal {O}(\epsilon )$ perturbation leads to $q_\Vert = 1+\epsilon q_1, \, q_1<0, \, q_\perp =\epsilon q_2$ and induces weak velocity perturbations $\epsilon \textbf{v}$, which may be superimposed on an $\mathcal {O}(1)$ base velocity.

It is advantageous to express the velocities through the stream function, $v_i=\varepsilon _{ij}\partial _j\Psi$, where $\varepsilon _{ij}$ is the 2D Levi-Civita antisymmetric symbol. Then $O(\epsilon )$ symmetric and antisymmetric rate of strain tensors $A^\pm$ are expressed as:

17 $$\begin{aligned} A^+&= \begin{pmatrix} \partial _1\partial _2 &  \textstyle \frac{1}{2}( \partial _2^2-\partial _1^2) \\ \textstyle \frac{1}{2}( \partial _2^2-\partial _1^2)&  - \partial _1\partial _2 \end{pmatrix} \Psi , \\\quad A^-&= \begin{pmatrix} 0 &  - \textstyle \frac{1}{2}( \partial _2^2+\partial _1^2) \\ \textstyle \frac{1}{2}( \partial _2^2+\partial _1^2) &  0 \end{pmatrix} \Psi . \end{aligned}$$

After omitting in the linearized hydrodynamic equation (15) the quadratic term due to weak deviations from the original alignment, the only remaining component of the antisymmetric stress is $-2\eta ^-_2 A^-_{21}$.

In an incompressible fluid, pressure is eliminated by applying the curl operator $\varepsilon _{ij}\partial _j$. Keeping for the antisymmetric term $k=2$ and $a=1$, this converts (15) into the equation of the stream function

18 $$\begin{aligned} \rho \partial _t \nabla ^2\Psi =\varepsilon _{ij}\partial _j [(\eta ^+\partial _k A^+_{ik}-2\partial _2\eta ^- A^-_{i2})]. \end{aligned}$$

In the last term, $q_a$ corresponding to the unperturbed alignment is set to unity and the corresponding index at $\eta ^-$ is omitted.

Turning to the dynamic equation of the order parameter with $\alpha$ set to unity, we have $\varrho =1+2\epsilon q_1<1$. Since the only leading order component of Eq. (12) is $\varpi _2= 2A^-_{21}$, it induces the $O(\epsilon )$ transverse component $q_2$. Using Eqs. (5)–(7), (12), (13), we obtain the linearized Eq. (13) in the explicit form

19 $$\begin{aligned} \partial _tq_1&= \Gamma [2\epsilon q_1 +K_1(\partial ^2_1 q_1 +\partial _1\partial _2 q_2) +K_2(\partial ^2_2 q_1 -\partial _1\partial _2 q_2], \\\partial _tq_2&= \Gamma [ K_1(\partial ^2_2 q_2 +\partial _1\partial _2 q_1) +K_2 (\partial ^2_1 q_2 -\partial _1\partial _2 q_1)]+2A^-_{21}. \end{aligned}$$

After expanding the stream function in the Fourier series

20 $$\begin{aligned} \Psi =\int \widehat{\Psi }(\textbf{ k})\textrm{e}^{\textrm{i}\mathbf { k\cdot x}}\textrm{d}^2 \textbf{ k} \end{aligned}$$

and expressing the components of the wave vector as $k_1=\kappa \cos \theta , \,k_2=\kappa \sin \theta$ (where $\theta =0$ corresponds to the unperturbed orientation of the order parameter and flow direction), the Fourier components of ${A}^\pm$ take the form

21 $$\begin{aligned} \widehat{\textbf{A}}^+&=- \textstyle \frac{1}{2}\epsilon \kappa ^2 \begin{pmatrix} \sin 2 \theta &  \cos 2 \theta \\ \cos 2 \theta &  - \sin 2 \theta \end{pmatrix}\widehat{\Psi }, \\\quad \widehat{\textbf{A}}^-&=- \textstyle \frac{1}{2}\epsilon \kappa ^2 \begin{pmatrix} 0 &  1\\ - 1 &  0 \end{pmatrix} \widehat{\Psi }= -\textstyle \frac{1}{2} \epsilon \kappa ^2\boldsymbol{\varepsilon }\widehat{\Psi }, \end{aligned}$$

and Eq. (18) reduces to

22 $$\begin{aligned} \rho \partial _t \nabla ^2\Psi =\varepsilon _{ij}\partial _i (\eta ^+\partial _k A^+_{jk}-2\eta ^- \partial _2A^-_{j2}), \end{aligned}$$

or, using (21) and canceling common powers of $\kappa$,

23 $$\begin{aligned} \rho \partial _t \Psi =-\textstyle \frac{1}{2} \kappa ^2( \eta ^+ -\eta ^- \sin ^2 2\theta ). \end{aligned}$$

Formally, this expression does not give a clear answer, as long as the values of the viscous coefficients are not specified. The standard symmetric viscosity should be positive, and stability is formally ensured when $\eta ^-<\eta ^+$, but it is reasonable to expect that the effect of antisymmetric viscosity be neither stabilizing nor destabilizing, which would require $\eta ^-$ to be purely imaginary. Under these conditions, the real part of the right-hand part of (23) testifies the decay of convective fluctuations due to the standard symmetric viscosity, while the imaginary part, originating in interaction between perturbations of nematic alignment and flow, causes the decay to be *oscillatory*, so that, e.g., fluctuations normal to the original alignment generate waves in the parallel direction before fading away. Decaying hydrodynamic oscillations should induce, through the coupling term (12) in Eq. (13), weak decaying oscillations of the order parameter.

A fully 3D analysis is facilitated in the case of an incompressible fluid by expressing its velocity through the *vector* analog of the stream function as $v_i=\varepsilon _{ijk}\partial _j\Psi _k$ with $\nabla \cdot \boldsymbol{\Psi }=0$ [23]. With $\Psi \sim {O}(\epsilon )$, the symmetric and antisymmetric strains are presented as

24 $$\begin{aligned} A^\pm _{il}= \epsilon (\varepsilon _{ijk}\partial _j\partial _i \pm \varepsilon _{ljk}\partial _j\partial _l)\Psi _k. \end{aligned}$$

Also in this case, pressure is eliminated by applying to Eq. (15) the curl $\varepsilon _{ijl}\partial _j$. This device is not so useful, as it does not reduce the number of equations but, as above, the leading-order system lacks destabilizing terms.

What is the cause of spurious instabilities detected in Ref. [17]? The answer is simple. This computation, carried out in the framework of the EL theory, *included* a linear feedback of nematic orientation on hydrodynamics carried by the “passive stress” proportional to the “tumbling parameter” $\chi$ and dependent on the direction of weak perturbations. This feedback makes stability analysis far more involved, requiring computation of Routh–Hurwitz determinants (which would be problematic in the case of complex inputs) and, most importantly, induces instability in a certain range of $\chi$ at some perturbation angles in the absence of active inputs. These instabilities are realized as spurious when we recall, as has been already discussed in the preceding Section, that “tumbling parameters” are just artifacts serving to ensure required symmetries in the procedure based on Onsager’s relations, disconnected from physical reality.

## Conclusion

The suggested mechanism of interactions between changes of nematic alignment and flow is not dependent on proximity to equilibrium and follows naturally from the symmetry between rotation of nematic alignment and flow vorticity, which, unlike its common treatment, should induce antisymmetric stress.

The established way to derive alignment-flow interactions through Onsager’s reciprocal relations, aspiring to be model-independent, turns out to be faulty, as it allows for spurious instabilities, which can be traced to non-physical “tumbling parameters” parameters formally required for the consistency of derivations in the established formalism.

The wide and protracted application of the established nematodynamic theory may invalidate the results of certain derivations and simulations on various scales, from molecular to macroscopic, so that they would need revision. Flow-alignment interactions and the way they are affected by activity are likely to be specific in different applications, especially biologically related, and require further deep insights.

## Data Availability

No data are associated with the manuscript.
